# Supplementary material for: MetaRibo-Seq measures translation in microbiomes
Source: Nat Commun. 2020 Jun 29;11:3268. doi: 10.1038/s41467-020-17081-z (PMC7324362; doi:10.1038/s41467-020-17081-z)
Supplement: Supplementary file 10 — Supplementary Data 7 [file 41467_2020_17081_MOESM10_ESM.zip › File2/Confidence_High_Taxonomy/290605_out.krona.html]

Javascript must be enabled to view this page.

members
magnitude
magnitudeUnassigned
count
unassigned
taxon
rank

290605\_out

75

superkingdom
75
2

976
75
phylum

200643
75
class

order
75
171549

815
75
family

75
genus

SRS016897\_contig\_number\_1400SRS020394\_contig\_number\_10134SRS047433\_contig\_number\_4437SRS049712\_contig\_number\_4503SRS050925\_contig\_number\_19364SRS062701\_contig\_number\_4214SRS144183\_contig\_number\_19472SRS148091\_contig\_number\_1930SRS148721\_contig\_number\_38745
9
816

64
species

SRS011084\_contig\_number\_19148SRS013800\_contig\_number\_19228SRS013965\_contig\_number\_36361SRS014613\_contig\_number\_805SRS014683\_contig\_number\_9067SRS015369\_contig\_number\_8541SRS015486\_contig\_number\_contig-100\_475.20317SRS015578\_contig\_number\_19167SRS015794\_contig\_number\_497SRS015890\_contig\_number\_6130SRS016267\_contig\_number\_147SRS018133\_contig\_number\_3438SRS018817\_contig\_number\_675SRS018984\_contig\_number\_4894SRS019030\_contig\_number\_16050SRS019496\_contig\_number\_14147SRS021484\_contig\_number\_3522SRS021948\_contig\_number\_32006SRS022609\_contig\_number\_42980SRS024388\_contig\_number\_8844SRS045004\_contig\_number\_25221SRS045645\_contig\_number\_945SRS045713\_contig\_number\_10822SRS046068\_contig\_number\_4186SRS046712\_contig\_number\_2219SRS046717\_contig\_number\_7074SRS049959\_contig\_number\_36122SRS053214\_contig\_number\_contig-100\_630.131385SRS058770\_contig\_number\_22324SRS063040\_contig\_number\_44198SRS063370\_contig\_number\_10999SRS064276\_contig\_number\_39431SRS064557\_contig\_number\_4051SRS065504\_contig\_number\_35457SRS075984\_contig\_number\_353SRS076804\_contig\_number\_17918SRS077127\_contig\_number\_17SRS077294\_contig\_number\_14219SRS077730\_contig\_number\_contig-100\_713.118818SRS098061\_contig\_number\_contig-100\_1884.74061SRS098881\_contig\_number\_3492SRS1041036\_contig\_number\_210SRS1041132\_contig\_number\_6628SRS1041142\_contig\_number\_5499SRS1041144\_contig\_number\_2646SRS1041145\_contig\_number\_contig-100\_18.196036SRS104311\_contig\_number\_28462SRS104327\_contig\_number\_2849SRS104693\_contig\_number\_2736SRS105082\_contig\_number\_22709SRS1055038\_contig\_number\_2597SRS1055043\_contig\_number\_21781SRS1055067\_contig\_number\_16481SRS1055095\_contig\_number\_893SRS1055099\_contig\_number\_11171SRS140513\_contig\_number\_7593SRS142542\_contig\_number\_19410SRS143372\_contig\_number\_779SRS143876\_contig\_number\_23101SRS147272\_contig\_number\_contig-100\_173.25739SRS149075\_contig\_number\_8767SRS893170\_contig\_number\_11630SRS893382\_contig\_number\_contig-100\_216.26597SRS893383\_contig\_number\_contig-100\_4010.175674
818

1
species

SRS148253\_contig\_number\_3452
1263054


SRS019787\_contig\_number\_1511
817
species
1
